# Comprehensive analysis of 66 complete molluscum contagiosum virus (MOCV) genomes: characterization and functional annotation of 47 novel complete MOCV genomes, including the first genome of MOCV genotype 3, and a proposal for harmonized MOCV genotyping indexing

**DOI:** 10.1128/mbio.02224-23

**Published:** 2023-11-10

**Authors:** Tomaž Mark Zorec, Erik Alm, Maria Lind Karlberg, Reza Advani, Lea Hošnjak, Mario Poljak

**Affiliations:** 1 Laboratory for Molecular Microbiology and Slovenian HIV/AIDS Reference Center, Institute of Microbiology and Immunology, Faculty of Medicine, University of Ljubljana, Ljubljana, Slovenia; 2 Department of Microbiology, Public Health Agency of Sweden, Solna, Sweden; Virginia Tech, Blacksburg, Virginia, USA

**Keywords:** molluscum contagiosum virus, molluscum contagiosum virus genotyping, complete genome sequencing, genomic diversity, recombination, complete genome restriction mapping, *de novo* assembly, genome annotation, molluscipoxvirus harmonization

## Abstract

Four molluscum contagiosum virus (MOCV) genotypes (MOCV1–4) and four subtype variants (MOCV1p, MOCV1va, MOCV1vb, and MOCV1vc) were partially characterized using restriction enzyme profiling in the early 1980s/1990s. However, complete genome sequences of only MOCV1 and MOCV2 are available. The evolutionary pathways of MOCV genotypes and subtype variants with unavailable sequences remain unclear, and also whether all MOCV genotypes/subtype variants can be reliably detected and appropriately categorized using available PCR-based protocols. We *de novo* fully characterized and functionally annotated 47 complete MOCV genomes, including two putative non-MOCV1/2 isolates, expanding the number of fully characterized MOCV genomes to 66. To ascertain the placement of any putative novel MOCV sequence into the restriction profiling typing scheme, we developed an original framework for extracting complete MOCV genome sequence-based restriction profiles and matching them with reference restriction profiles. We confirmed that two putative non-MOCV1/2 isolates represent the first complete genomes of MOCV3. Comprehensive phylogenomic, recombination, and restriction enzyme recognition site analysis of all 66 currently available MOCV genomes showed that they can be agglomerated into six phylogenetic subgroups (PG1–6), corresponding to the subtype variants from the pioneering studies. PG5 was a novel subtype variant of MOCV2, but no PGs corresponded to the subtype variants MOCV1vb or MOCV4. We showed that the phylogenetic subgroups may have diverged from the prototype MOCV genotype lineages following large-scale recombination events and hinted at partial sequence content of MOCV4 and direction of recombinant transfer in the events that spawned PG5 and the yet undetected subtype variant MOCV1vb.

**IMPORTANCE:**

Four molluscum contagiosum virus (MOCV) genotypes (MOCV1–4) and four subtype variants were partially characterized using restriction enzyme profiling in the 1980s/1990s, but complete genome sequences of only MOCV1 and MOCV2 are available. The evolutionary pathways whereby genotypes/subtype variants with unavailable sequences emerged and whether all MOCVs can be detected using current diagnostic approaches remain unclear. We fully characterized 47 novel complete MOCV genomes, including the first complete MOCV3 genome, expanding the number of fully characterized genomes to 66. For reliably classifying the novel non-MOCV1/2 genomes, we developed and validated a framework for matching sequence-derived restriction maps with those defining MOCV subtypes in pioneering studies. Six phylogenetic subgroups (PG1–6) were identified, PG5 representing a novel MOCV2 subtype. The phylogenetic subgroups diverged from the prototype lineages following large-scale recombination events and hinted at partial sequence content of MOCV4 and direction of recombinant transfer in the events spawning PG5 and yet undetected MOCV1vb variant.

## INTRODUCTION

Molluscum contagiosum (MC) is one of the 50 most prevalent diseases worldwide (1–3). MC manifests as umbilicated skin papules, limited in size (<5 mm) and number (<20), which gradually regress within weeks or months, often not requiring treatment, but with less favorable clinical outcomes in immunocompromised individuals, in whom clinical intervention is needed more often due to larger portions of inflicted skin surfaces and longer disease persistence (4–9).

Molluscum contagiosum virus (MOCV), the causative agent of MC, is the last still naturally circulating exclusively human-infecting poxvirus since the eradication of smallpox (6). Until the characterization of equine molluscum contagiosum-like virus (EMCLV) (10), MOCV had been the only member of the unique taxonomic genus *Molluscipoxvirus*. To date, only two molecular genotypes of MOCV, which are two distinct molecular species, have been characterized at the complete genome level and classified as MOCV1 and MOCV2 (11–14). However, a total of four MOCV types (MOCV1-4) and four subtype variants of MOCV1 (MOCV1va, MOCV1vb, and MOCV1vc) have been identified with complete genome restriction profiling in early molecular epidemiological studies of MOCV in the 1980s and 1990s (15–20).

The available nucleotide sequence data allowed the development of molecular assays for the detection of MOCV and classification of its molecular types based on polymerase chain reaction (PCR), quantitative PCR, and/or the sequencing of various fragments, including the nucleotide sequence of the MOCV gene *MC021L* (21–24). Due to their relative simplicity, high specificity, and sensitivity, these PCR- and/or sequencing-based MOCV detection and genotyping protocols have since been the test of choice for diagnostic purposes and molecular-epidemiological surveys (2, 25–28). Because MOCV1 and MOCV2 have been the only two MOCV genotypes supported by publicly available sequence data, it is unclear whether these assays can detect or differentiate between MOCV genotypes other than MOCV1/2. In fact, due to the lack of data, it has been unclear whether non-MOCV1/2 genotypes still exist or are circulating among humans, and where and in what type of evolutionary relationships they are with MOCV1/2.

Here, we characterize 47 novel complete MOCV genome sequences, including 38 novel genomes of MOCV1, 9 novel genomes of MOCV2, and 2 complete genomes of putative non-MOCV1/2 isolated from two immunocompetent Swedish children in 2014 and 2016 (29). All 47 genomes and genome drafts were assembled *de novo,* functionally annotated, and screened for the most relevant mutations. To determine which of the restriction profiling-based MOCV types correspond to putative non-MOCV1/2 genomes, we developed an original theoretical framework for matching sequences of complete MOCV genomes and genome drafts to literature reference restriction enzyme profiles of the MOCV1–4 types and their subtype variants. The Swedish sequences turned out to be the first complete genomes of MOCV3. Further comprehensive phylogenomic and recombination analyses carried out on all 66 currently available MOCV genomes (64 complete genomes and 2 complete genome drafts) showed that they can be agglomerated into six phylogenetic subgroups (PG1–6), which correspond to the subtype variants from the early pioneering studies. PG5 represented a novel subtype variant of MOCV2, but no PGs corresponded to the subtype variants MOCV1vb or MOCV4. We also showed that the phylogenetic subgroups may have diverged from the prototype MOCV genotype lineages following large-scale recombination events and hinted at partial sequence content of MOCV4 and direction of recombinant transfer in the events that spawned PG5 and the yet undetected subtype variant MOCV1vb.

## RESULTS AND DISCUSSION

### Phylogenomic analysis revealed the presence of a third MOCV genotype

**Fig 1:**
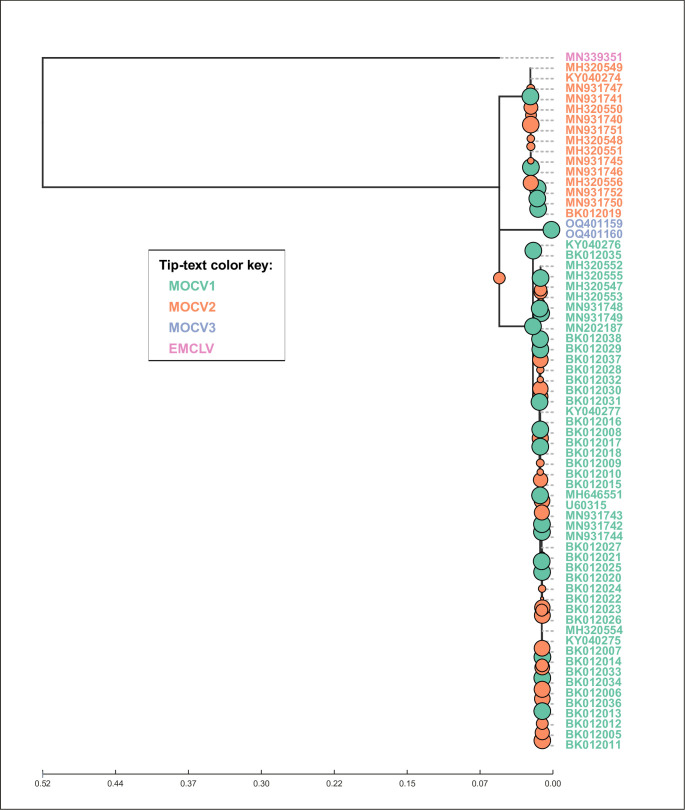
The *Molluscipoxvirus* phylogenomic tree. The phylogenomic tree was constructed based on the multiple sequence alignment of complete *Molluscipoxvirus* genomes available in GenBank, including MOCV and EMCLV. Phylogenetic reconstruction was produced with IQ-Tree (30, 31) using the substitution model GTR + F + R4. Node support values, depicted by the size of the green and orange node markers, were estimated using UFBootstrap (32). Nodes with saturated UFBootstrap scores (100/100) are colored green. The tree was rooted at the branch with the tip representing EMCLV (GenBank accession number MN339351) using ete3 Toolkit (33) and visualized using the Python (v3.8) module Toytree. Tree tips have been colored according to the MOCV genotype or species (in the case of EMCLV).

After collecting complete MOCV genome sequences from the GenBank database and reconstructing novel complete MOCV genomes from sequencing reads, we performed phylogenomic reconstruction of the genus *Molluscipoxvirus,* which included all 66 available complete MOCV genome sequences and the complete genome sequence of EMCLV (MN339351) (10). The genus-scale phylogenomic tree suggested a clear distinction between EMCLV and MOCV (Fig. 1). The clade corresponding to MOCV had three stems, one for each of the known genotypes: MOCV1 (with the prototype sequences: U60315, KY040275, KY040276, and KY040277) and MOCV2 (prototype sequence: KY040274). The third MOCV stem held two novel MOCV sequences, OQ401159 and OQ401160.

The sequences OQ401159 and OQ401160 are presently the only two complete genome sequences of MOCV obtained from Swedish patients. They originated from giant *mollusca* from two immunocompetent children from Sweden, dated 2014 and 2016. Both cases were reported previously in Hammarin et al. (29), where the authors used un-scaffolded draft viral genomes and concluded that the virus belonged to an unknown or previously unsequenced type of MOCV. Here, these sequence read data sets were reassembled and scaffolded to the level of complete genome scaffolds and analyzed further. A short gap remains in the sequence OQ401159 at position 70 kb, unlike OQ401160, which has been characterized completely, this sequence may still be considered as a complete genome draft.

The sequences OQ401159 and OQ401160 appeared closely related to each other (Hamming similarity: 0.998) and showed a similar degree of divergence from the common ancestor of MOCV as MOCV1 and MOCV2 (Fig. 1), with Hamming similarities to the prototype sequences of MOCV1 (U60315) and MOCV2 (KY040274) of 0.92 and 0.93, respectively. Inter-genotype Hamming similarities of MOCV have previously been shown to range below 0.97, whereas intra-genotype pairwise Hamming similarities ranged well above this threshold (14). Collectively, this confirms the idea that the sequences OQ401159 and OQ401160 should be considered a non-MOCV1/2 genotype, as has already been pointed out by Hammarin et al. (29).

### Phylogenomic MOCV genotypes are equivalent to complete genome restriction-profiling-based MOCV types

To determine whether the identified non-MOCV1/2 genotype, represented by the two novel sequences, OQ401159 and OQ401160, had been encountered before and, if so, which of the restriction-profiling-based MOCV types it corresponded to, we developed a theoretical framework for aligning restriction-profiling-based MOCV types with sequence-based genotypes. We searched the complete MOCV genome sequences for restriction enzyme (*BamHI*, *HindIII*, and *ClaI*) recognition sites and compared them to the reference restriction maps derived from the reports in Nakamura et al. (19) and Porter and Archard (18).

**Fig 2:**
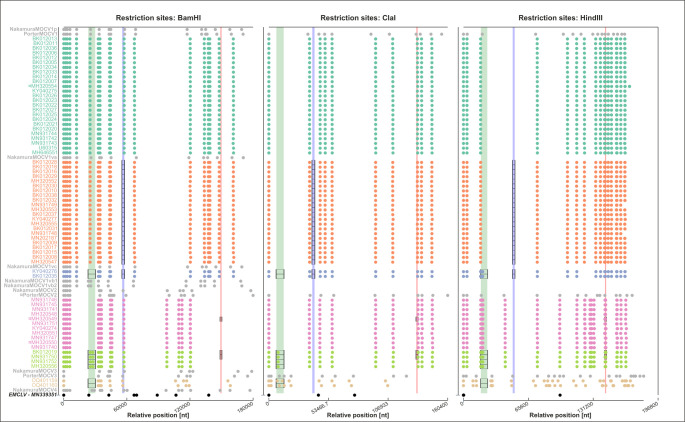
*BamHI*, *ClaI*, and *HindIII* restriction maps based on relative restriction site coordinates in complete MOCV genome sequences plotted alongside literature reference restriction maps of the MOCV subtypes MOCV2-4 and the MOCV1 subtype variants MOCV1p, MOCV1va, MOCV1vb, and MOCV1vc (18, 19), and the restriction map derived from the sequence MN339351 representing EMCLV (10). Relative sequence coordinates denote the distance from the first restriction site. In the case of *HindIII* restriction maps for the sequences MH320549 and MH320550 and the reference profile PorterMOCV2, the relative coordinates denote the distance from the second *HindIII* restriction site, and in the sequence MH320554 from the third *HindIII* restriction site; the restriction map labels are marked with * and **, respectively. Restriction map labels are colored according to the MOCV phylogenetic subgroup, and the reference restriction maps are drawn in black/gray. Relative positions of the recombinant sequence segments are illustrated using semi-transparent colored bands, and the actual recombinant segments are marked with black rectangles. Recombinant sequence segments were found using silhouette analysis, and breakpoints were set using Recombination Program 5 (34) and confirmed with phylogenetic contrast, as described previously (14). The restriction maps were visualized using the Python 3.8 module Toyplot.

On first inspection, the restriction profiles derived from sequence data were well-conserved (Fig. 2): shifts in relative positions, trimmed to start with the first identified restriction site in each sequence, of contextually equivalent restriction sites were small, and sequences of the same genotype presented with similar numbers and (relative) genomic positions of restriction sites. Most importantly, restriction maps of MOCV1 could easily be distinguished from MOCV2 at the level of visual inspection. On the other hand, shifts were clearly noticeable when comparing sequence-derived restriction maps and literature-derived restriction maps, although the sequence-based restriction maps could still be unambiguously matched with literature-derived restriction maps of the equivalent MOCV genotypes (Fig. 2). These greater shifts along the sequence-based/literature-derived contrast might have arisen from factors such as poor resolution of restricted fragments of equivalent sizes and lack of precision in fragment size determination. These values were read and determined by the authors of the original studies based on reference fragment size ladders (18, 19).

The restriction maps derived from sequences belonging to the phylogenomic genotypes MOCV1 and MOCV2 corresponded most closely to literature-based restriction patterns of the MOCV1 and MOCV2 types, respectively. This confirms that sequence-based phylogenetic genotyping of MOCV and restriction enzyme map-derived determination of MOCV types are effectively equivalent and justify the interchangeable use of the terms MOCV types and MOCV genotypes.

### OQ401159 and OQ401160 are the first sequences and complete genome drafts of MOCV3

The restriction maps encoded by the sequences OQ401159 and OQ401160 most closely resembled the reference maps of MOCV3 (Fig. 2): the two sequences are the first complete genomes of the genotype MOCV3. In this case, the restriction site position shifts were clearer, both along the sequence/literature contrast, as well as between the two sequence-derived restriction maps. Shifts between restriction maps along the sequence-based/literature-derived contrast may be explained by the same types of bias as discussed above; in contrast, position shifts between the two sequence-derived restriction maps likely did arise from errors related to *de novo* assembly and scaffolding. In fact, the sequence OQ401159 contains a short region at position 70 kb, which could not be reassembled confidently and was arbitrarily filled with 100 *N*-characters, while OQ401160 could be confidently characterized along the entire length of the genome. On the one hand, restriction sites in the gapped region of OQ401159 could not be identified, and on the other, the positions of the restriction sites further downstream from this region appeared shifted in relation to the restriction sites in OQ401160, explaining restriction-site position variation between these two sequences.

### MOCV3 genomes encode between 179 and 180 out of 182 core MOCV genes

All genomic features found in the newly assembled complete and draft complete MOCV genome sequences have been included as genome annotation features and submitted to the NCBI GenBank database along with the genome sequences, including the two MOCV3 genomes, OQ401159 and OQ401160.

The draft genome OQ401159 has one sequence gap, a region that could not be confidently reassembled with the available data, arbitrarily filled with 100 *N*-characters located at position 70,034 bp. The gap contextually corresponded to the region following the gene *MC053.2R* and truncated the termination of the gene *MC054L*.

The genomes, OQ401159 and OQ401160, contained a total of 483 and 442 open reading frames (ORFs), respectively, which could encode genes based on at least one level of inference: similarity to MOCV genes, similarity to non-MOCV poxviral genes, or *de novo* inference according to Prokka/Prodigal. Of these, 262 and 222 ORFs corresponded to 179 and 180 of the 182 MOCV genes described in Senkevich et al. (12) and 174 ORFs mapped to non-MOCV poxviral genes that were common to both draft genomes (Table S4). These common ORFs matched genes from 76 different GenBank sequences and from 21 poxviral genera and 49 poxviral species (see Table S4 for details). The most common ORFs matched orthopoxviral genes, most frequently (20 matching taxa) those encoded by Cowpox and Vaccinia viruses, followed by genes from the genera *Parapoxvirus* (10 matching taxa) and *Crocodylipoxvirus* (8 matching taxa). The common ORFs ranged in length between 77 and 1,367 bp (mean: 247.01 bp; percentiles: p5: 86.00 bp, p25: 119.00 bp, median: 189.50 bp, p75: 287.00 bp, p95: 670.00 bp) and may be novel MOCV genes encoding potentially novel peptides and proteins (details in Table S4).

A total of 32 (11 inferred *de novo*) and 37 (15 inferred *de novo*) ORFs matched non-MOCV poxviral genes but were unique to either OQ401159 or OQ401160, respectively.

The mismatches in the numbers of MOCV genes and the numbers of corresponding ORFs hinted at fragmentation due to the introduction of stop codons. Some of these stop codons may have been introduced by sequencing and/or assembly errors but seeing the same gene interruption in two independent sequences compellingly suggests that the interruption is of biological origin. Perhaps the proteins in question consist of multiple independent domains that can then further aggregate into multi-part complexes if encoded in separate ORFs. On the other hand, these could be multifunctional proteins with different domains that can function and be expressed independently or as peptides. In some cases, the interruptions could be leveraged by assuming frameshifts that would avoid the stop codons, but we wondered about the sensibility of doing so, first because such assumptions are arbitrary and, second, because it becomes difficult to determine which parts of the ORF are part of the annotated gene and which are not because we did not find unambiguous intron signatures. We decided to leave the annotations in the form of ORFs annotated as coding sequence regions (CDS features) and to allow multiple CDS features per gene. Core MOCV genes and the numbers of corresponding CDS features are listed in Table S4.

The following 15 MOCV genes had equivalent interruption patterns in both draft genomes: *MC009.2R, MC010R, MC014R, MC018L, MC035R, MC036R, MC040.1L, MC052R, MC055R, MC092R, MC107L, MC144R, MC150R, MC152.1R, and MC154R*. Of note, *MC035R* encodes a homolog of the Variola B22 family protein, which has been shown to inactivate/prevent the activation of T cells in culture and animal models (35, 36).

The genomes OQ401159 and OQ401160 commonly lacked the MOCV genes *MC053.1R* and *MC149.1R*, and the sequence OQ401159 further lacked the MOCV genes *MC017.1L* and *MC020L*. Neither of the missing genes has a known function related to immune evasion or viral replication or shows similarity to other known proteins. Other than that, all likely core MOCV genes discussed in Senkevich et al. (12) were present in the sequences of MOCV3.

### MOCV genomes could be aggregated into phylogenetic subgroups with conserved restriction profiles

**Fig 3:**
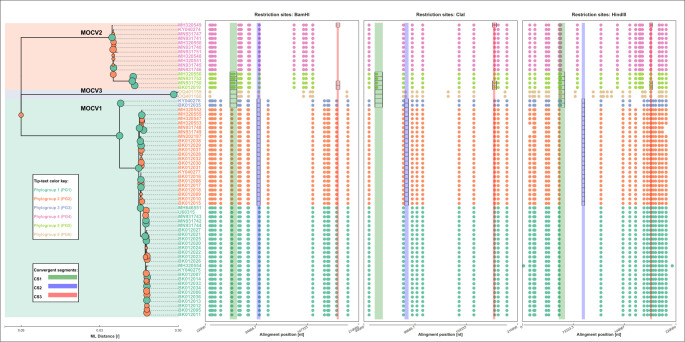
Phylogenomic tree of complete MOCV genome sequences (left) and *BamHI*, *ClaI*, and *HindIII* restriction maps based on restriction site positions in the multiple complete MOCV genome sequence alignment (right). Left: the phylogenomic tree was constructed based on the multiple sequence alignment of complete *Molluscipoxvirus* genomes using IQ-Tree (30, 31) and the substitution model GTR + F + R4; node support values, depicted by the size of the green and orange node markers, were estimated using UFBootstrap (32). Nodes receiving saturated UFBootstrap scores (100/100) are colored green. The tree was rooted at the branch with the tip representing EMCLV (GenBank accession number MN339351). The tree was visualized using the Python (v3.8) module Toytree. The area on the tree is colored according to the genotype of the sequences embedded within; tree tips are colored according to the proposed MOCV phylogenetic subgroups. Right: the restriction maps were drawn by searching the complete MOCV genome sequences for restriction enzyme recognition patterns and transforming their genomic coordinates onto multiple sequence alignment coordinates. The restriction pattern recognition site was made possible by the Python 3.8 module Re, and the maps were visualized using the Python 3.8 module Toyplot. The samples are organized in the same order as in the phylogenomic tree (left), and plot-marker colors stand for the proposed phylogenetic subgroups of the MOCV genomes. Alignment positions of the recombinant segments are depicted using semi-transparent colored bands, and the actual recombinant segments are marked with black rectangles. Recombinant sequence segments were found using silhouette analysis, and breakpoints were set using the Recombination Program 5 (34) and confirmed with phylogenetic contrast, as described previously (14).

The part of the *Molluscipoxvirus* phylogenomic tree encoding phylogenomic relationships among MOCV sequences was inspected in greater detail (Fig. 3, left). In addition to the divergence between genotypes, the tree revealed further divergence at the intra-genotype level. This second-level divergence allowed further aggregation of MOCV genomes into six phylogenetic subgroups/phylogroups: PG1 to PG6. PG1, PG2, and PG3 were child clades of MOCV1, PG4 and PG5 were child clades of MOCV2, and PG6 corresponded to MOCV3 (Fig. 3, left). Annotating the tree-tips and plotting the tree side by side with the aligned sequence-derived restriction maps revealed nearly perfect conservation of restriction enzyme recognition sites within the phylogenetic subgroups identified (Fig. 3, right). Specifically, for the enzyme *BamHI*, the MOCV1 restriction maps in PG2 diverged from PG1 by one restriction site found at the alignment position 87 kb, and PG3 by another restriction site at the alignment position 57 kb. *BamHI* restriction maps of PG4 and PG5 diverged from one another by two restriction sites found at the alignment positions 57 and 58 kb. Precise genomic coordinates in alignment and genomic coordinates relative to the MOCV1 reference sequence U60315 of the identified restriction sites are available in Table S3.

### Phylogenetic subgroups are equivalent to the restriction-profiling subtype variants

In addition to defining MOCV types based on restriction profiles obtained following enzymatic digestion of complete MOCV genomes, the pioneering molecular epidemiological studies of MOCV also recognized a set of restriction profiles that diverged slightly from the prototype profile of MOCV1. These profiles were addressed as belonging to subtype variants MOCV1va, MOCV1vb, and MOCV1vc (19). As in the case of MOCV genotypes and types, comparing the conserved restriction maps of the identified phylogenetic subgroups to the literature-derived restriction maps of the subtype variants revealed that the two methods of classification convey equivalent information: the subgroups PG1, PG2, and PG3 corresponded, respectively, to the prototype variant, MOCV1p, and the subtype variants, MOCV1va and MOCV1vc, defined in Nakamura et al. (19) (Fig. 2).

### MOCV lineages harboring recombination at *RS1*, *RS2*, and/or *RS3* are spread worldwide

**Fig 4:**
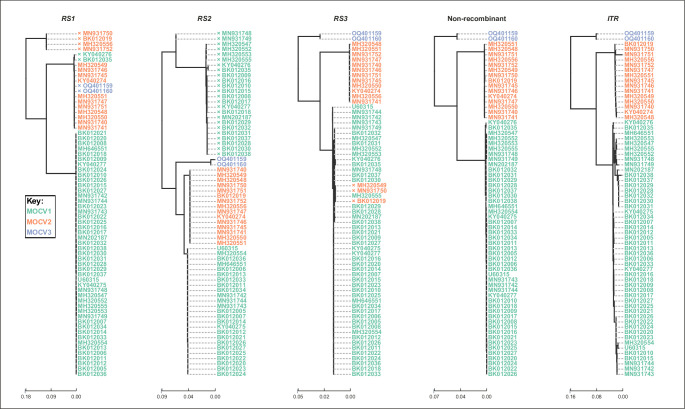
Phylogenetic trees of the MOCV genome sequence segments that suggest recombination (*RS1–3*), the concatenated non-recombinant parts of the MOCV genomes (non-recombinant), and the inverted terminal repeats (*ITRs*). Tree-tips are colored according to MOCV genotype. The decoupled sequence regions were analyzed using IQ-tree (30, 31) to produce independent phylogenetic trees using the substitution model GTR + F + R4 and UFBootstrap node support values (32). Trees were rooted at the midpoint using the ete3 Toolkit (33) and visualized using the Python 3.8 module Toytree.

We searched the newly updated collection of complete MOCV genomes for sequence segments showing signs of recombination. We used a sliding kernel-based analysis of silhouette coefficients to find candidate recombinant sequence segments (Fig. S1) and RDP5 (34) to pinpoint the precise genomic locations of the putative recombination breakpoints. Phylogenetic assessment was used for confirmation, specifically incongruence between trees of the recombinant segments (*RSs*) versus the complete genome phylogeny. Our analysis revealed three *RSs*, enumerated *RS1–3* (Fig. 4), whose overall genomic positions corresponded with those reported previously (13, 14). Genomic coordinates of convergent sequence segments are available in Table S3. Several genomes were recombinant at either one of the *RSs* (Fig. 4) and originated in Spain, Slovenia, the United States, Australia, and Sweden (Table S1), which compellingly shows that the recombinant lineages are persistently spread worldwide.

Searching the NCBI nt database for non-MOCV *RS*-similar nucleotide sequences with discontiguous megablast revealed no significant hits at relevant sequence identity and query cover levels, >80.00% and >50.00%, respectively. This, as well as the worldwide spread of recombinant lineages, speaks in favor of singular ancestral gene acquisition events in each *RS* and recombinant transfer between co-infecting MOCV genotypes, rather than the lineages having received the *RS* from non-MOCV entities post divergence from a common ancestor. The worldwide spread of recombinant MOCV lineages suggests a limited number of structural/evolutionary endpoints of the genes encoded by the *RS*s and demonstrates a certain degree of modularity of these genes; for example, the optimal state at one of the *RS* may be independent of which one of the attainable optima the genome assumes at any other *RS*.

The observation that recombinant sequence segments in MOCV genomes co-localize with regions encoding genes that are part of the virus-host interactome and the idea that this may be related to exploring various evolutionary optima attained by the separate lineages—MOCV genotypes—have been discussed previously (13, 14). *RS1* co-localizes with *MC035R,* an MOCV homolog of the B22 family proteins (35, 36), *RS2* co-localizes with the encodings of MOCV homologs of the human interleukin-18 binding protein, which can antagonize the production of interferon-gamma (*MC053L, MC054L, and MC056L*), and *RS3* with a MOCV CC-chemokine antagonist (*MC148R*) (37–41). It seems reasonable that these genes got acquired at some point after the onset of the specific virus-host niche adaptation by some type of gene capture. Homologs of MC035R appear in representatives of multiple chordipoxvirinal genera, among others *Orthopoxvirus*, *Crocodylipoxvirus,* and *Molluscipoxvirus* (10, 12, 35, 42). *MC053R, MC054R, MC056R,* and *MC148R* appear to have been introduced to MOCV only after the divergence from the common ancestor of MOCV and EMCLV (10). It is possible that the acquired gene sequences drifted apart to multiple homologous local optima as MOCV lineages diverged through time. On the other hand, as reviewed previously (37–43), novel *trans* recombinant MOCV types may have formed by homologous DNA recombination when the divergent lineages met again during co-infection.

### Phylogenetic placement of MOCV3 alongside MOCV2 at *RS1* raises questions about the direction of the recombinant transfer

*RS1,* first identified by López-Bueno et al. (13), has previously been shown to have been affected by at least two recombination events, RS1.E1 and RS1.E2 (14). RS1.E1, in the sequences KY040276 and BK0120035, appears to have been transferred to MOCV1 from MOCV2, and RS1.E2 appears to have been input to MOCV2 from an unknown source, most likely a co-infecting MOCV of a different genotype (Fig. 4). Our results suggest a novel recombination event at *RS1* (RS1.E3) concerning the two novel genomes of MOCV3, OQ401159 and OQ401160. These are the only sequences of MOCV3 to date; with the data presently available, it may also be that the clade in the *RS1* tree, embedding the sequences harboring RS1.E1 and the putative RS1.E3, is an ancestral “elder” clade, which the other lineages and genotypes diverged from; this scenario would explain the phylogenetic situations in Fig. 4 with only two recombination events instead of three.

The non-recombinant phylogenomic tree in Fig. 4 suggested that the common ancestor of MOCV1, MOCV2, and MOCV3 predated the common ancestor of MOCV1 and MOCV2; this is incongruent with Fig. 2, which suggested contemporaneous common ancestry of the three genotypes. This discrepancy arose due to different rootings: the phylogenomic tree in Fig. 2 was rooted using the EMCLV genome as an outgroup, whereas the non-recombinant phylogenomic tree in Fig. 4 was rooted at the midpoint. All trees in Fig. 4 were rooted at the midpoint, and all five trees may have been affected by a similar “whose ancestor was first”-type bias at their bases. The alternative hypothesis regarding the number of recombinant events in *RS1* (discussed in the previous paragraph), and in fact, the entire discussion around it, could be a direct consequence of this bias.

### Recombinant sequence segments co-localized with restriction enzyme recognition sites that are specific for differentiating between the proposed phylogenetic subgroups

We noted that harboring a recombination at *RS1* or *RS2* had a drastic impact on the sequences’ placement in the phylogenomic tree (Fig. 3, left). In fact, the sequences harboring RS2.E1 belong to the phylogroup PG2, the sequences harboring both RS1.E1 and RS2.E1 belong to PG3, and the sequences harboring RS1.E2 belong to PG5. Overlaying genomic coordinates of the recombinant sequence segments with the locations of restriction enzyme recognition sites revealed that several of the restriction sites were part of the recombinant sequence segments (Fig. 3, right), and for the most part, the restriction site patterns that were nested within the recombinant segments were also characteristic of the phylogenetic subgroups. The recombination RS1.E1 coincided with the loss of a *BamHI* restriction site and the gain of a *HindIII* restriction site at the alignment positions 54.5–55.5 kb in the sequences belonging to the phylogroup PG3 and the genotype MOCV1 (Fig. 3, right). RS2.E1 resulted in the loss of a *BamHI* restriction site at the alignment position 88.3 kb and affected the sequences belonging to the MOCV1 phylogenetic subgroups PG2 and PG3.

### Unclear origin of RS1.E2 hints that MOCV4 and MOCV1vb are out there, waiting to be found (again)

Expectedly, due to the phylogenetic placements of the MOCV1 sequences belonging to PG3 in Fig. 4, the loss and gain pattern of *BamHI* and *HindIII* restriction sites at *RS1* matched the restriction pattern otherwise seen in the bulk of MOCV2 sequences (Fig. 3, right). On the other hand, the MOCV2 sequences belonging to the phylogroup PG5 also harbored a recombination at *RS1,* RS1.E2 (Fig. 4, *RS1*), which resulted in the gain of two novel *BamHI* restriction sites and one novel *ClaI* restriction site at the alignment positions 55 and 57 kb, and 54.5 kb, respectively. Rather than convergence with other known sequences of MOCV, RS1.E2 is reflected in the substantial divergence of PG5 sequences from all other MOCV sequences, obscuring the information about its recombinant origin.

Figure 2 revealed a striking resemblance between the *RS1* restriction patterns of PG5 sequences, the literature-derived restriction maps of the type MOCV4, and the subtype variant MOCV1vb, which have so far only been reported in a single study (19), two *BamHI* restriction sites and one *ClaI* restriction site. This led us to speculate that RS1.E2 originated from a recombinant donor of the genotype MOCV4. Although we found no sequences that corresponded to the subtype variant MOCV1vb, the presence of the twin *BamHI* restriction site, co-localizing with *RS1*, might also suggest a recombinant nature of the lineage, spawned by a so-far undetected recombination event, RS1.E4, affecting the genomes of MOCV1 and possibly of a novel phylogenetic subgroup.

There is also a striking resemblance between Nakamura’s MOCV4 and PG5 along the entire *BamHI* restriction map. The only differences, non-amendable by slightly shifting the restriction sites’ positions, due to the possibility of poor fragment-size resolution in the old report, are three restriction sites beyond the trimmed relative genomic position at 125 kb and an extra restriction site at around 60 kb (Fig. 2). It may be that MOCV4 is simply a subtype variant belonging to MOCV2, but this question cannot be answered until a complete genome corresponding to MOCV4 is obtained.

### Mutations in novel MOCV genomes

We cataloged the mutations found in the updated collection of the (nearly) complete MOCV genomes (called and annotated in reference to the MOCV1 sequence U60315; Table S5). Numbers of deletions (DELs), insertions (INSs), and point mutations (SNPs) ranged between 17, 15, and 101, and 465, 485, and 10,255, respectively. Sequences belonging to the genotype MOCV1 had up to 77 DELs, 73 INSs, and 1,169 SNPs, with the medians at 35 DELs, 35 INSs, and 259 SNPs. Non-MOCV1 genomes had at least 366 DELs, 281 INSs, and 8,229 SNPs, with the medians at 402 DELs, 303 INSs, and 8,439 SNPs. The MOCV3 genomes had the most mutations relative to the MOCV1 reference. We also recorded and plotted the pairwise sequence similarities between the updated collection of MOCV genomes as well as their GC proportions as histograms; in addition to the complete genome-based statistic, the statistics based on different sequence segments have been included (recombinant, ITR, and non-recombinant; Fig. S2 and S3).

### A newly identified mutation may hinder the specificity of the current PCR-based MOCV genotyping assays

Mutations present in all novel MOCV genomes were cataloged (Table S5), and the results are summarized in the previous section.

Trama et al. (23) and Hošnjak et al. (24) used a fragment of the MOCV gene *MC021L* for sensitive and specific real-time (RT) PCR-based detection of MOCV in both fresh-frozen and formalin-fixed, paraffin-embedded tissue specimens. For typing, in the framework of Trama et al. (23), the sequence content had to be read, whereas Hošnjak et al. (24) differentiated between MOCV1 and MOCV2 based on the presence of the point mutation U60315.27451T>C, which was detected using melting curve analysis.

To assess what kind of outcome would be recorded if MOCV3 were fed to the RT-PCR assay developed by Hošnjak et al. (24), we inspected the sequence fragment (U60315.27379–27528) used in the assay in further detail. The alignment showed that the assay could not differentiate between MOCV3 and MOCV2. MOCV3 was identical to MOCV2 in the region recognized by the forward primer (mutation U60315.27397A>G) and identical to MOCV1 in the region recognized by the reverse primer, and there were no mutations other than U60315.27451T>C in the conserved region around it, and that is covered by the oligonucleotide anchor and probe, which the assay uses for detecting the SNP. The region between the forward primer and the oligonucleotide anchor was identical to MOCV1 (U60315.27402–27421), and the region between the oligonucleotide probe and the reverse primer (U60315.27465–27506) had two novel SNPs: U60315.27495G>T and U60315.27499G>T.

Furthermore, the alignment showed the presence of a novel SNP at the assay’s discriminatory position, U60315.27451T>G, in seven novel MOCV1 sequences from the United States (BK012008–BK012010 and BK012015–BK012018). These sequences would most likely raise the same signal as MOCV2 in the RT-PCR assay developed by Hošnjak et al. (24). This RT-PCR assay has so far been the most convenient molecular assay that allowed both sensitive and specific detection of MOCV infection as well as MOCV genotyping in a single RT-PCR reaction. Our results showed that (i) the genomic position that it uses to discriminate between MOCV1 and MOCV2 does not allow discrimination between MOCV2 and MOCV3 and (ii) there is a sub-lineage of MOCV1 that has an SNP at this position and would be misclassified as MOCV2. Even though the assay will reliably detect MOCV, it requires improvements to account for the novel molecular species in terms of genotyping. The earlier PCR-based assay described in Trama et al. (23) used the same oligonucleotide primers for amplification as Hošnjak et al. (24), but it used amplicon sequencing to confirm the MOCV identity and determine the MOCV genotype. A similar approach can readily be used to detect all four currently known molecular variants of this amplicon (MOCV2, MOCV3, and both amplicon variants of MOCV1).

### A need for harmonized nomenclature of MOCV genotypes and for substantially more MOCV complete genome sequences

Misunderstandings related to MOCV-type nomenclature leading to diverging systematics have been addressed and, with efforts, leveraged in the past (18). It is sensible to build upon those efforts when addressing and appointing potentially novel MOCV genotypes. Assignment of MOCV genotypes using a phylogenomic complete genome sequence-based approach should follow the same indexing that was used for the assignment of MOCV types using restriction enzymes in early studies (18, 19, 44). Herein, we have demonstrated that complete MOCV genomes can effectively be matched and cross-referenced with the literature restriction maps described in early studies. In addition, we propose that MOCV genotype classification should be based on pairwise sequence similarity, preferentially at the level of complete genomes. In our study, complete genome-level intra-genotype pairwise similarity values started just above 0.99 and inter-genotype similarities ranged between 0.92 and 0.95 (Fig. S2; Table S2), suggesting the value of 0.98 is a suitable lower bound for complete genome level intra-genotype sequence similarity followed by complete genome-level phylogenetic assessment for confirmation. In cases of potentially novel molecular species of MOCV genotypes, the reference restriction profiles should be consulted to determine a harmonized genotype index. Once identified, a novel complete MOCV genome sequence with a restriction map closely resembling that of MOCV4 would be named MOCV4, but any novel MOCV genotype not resembling any of the previously encountered restriction maps would be eligible for the next consecutive genotype index, such as MOCV5, MOCV6, and so on.

This study clearly showed a limited current knowledge of the genetic heterogeneity of MOCV. Large-scale molecular-epidemiologic surveys and sequencing efforts are needed with the aim of obtaining as many complete MOCV genomes as possible across different geographic regions. Although sequencing each and every isolate obtained would be a favorable approach, in time- and resource-limited settings, an assay using RT-PCR to amplify the region targeted by Trama et al. (23) and Hošnjak et al. (24) followed by the sequencing of only PCR amplicons seems to be a suitable screening approach, followed by characterization of the complete MOCV genome of random samples as well as each isolate with a unique PCR amplicon sequence not recorded so far.

### Conclusions

We report a comprehensive phylogenetic, recombination, and restriction enzyme recognition site analysis of a total of 66 (49 novel) complete MOCV genomes, including 49 (38 novel) genomes belonging to the genotype MOCV1, 15 (nine novel) genomes of MOCV2, and the first two complete genomes of MOCV3. All sequences obtained *de novo* in this study were functionally annotated, screened for mutations and signs of recombination, and deposited in the relevant public databases. The updated collection of complete MOCV genomes included mutations that may hinder the accuracy of some PCR-based MOCV genotyping assays currently in use. To allow the classification of the novel non-MOCV1/2 genome sequences, we developed and validated a theoretical framework for matching sequence-derived restriction maps with the restriction mapping used to define MOCV subtypes in early molecular-epidemiological studies of MOCV. Using this framework, we undoubtedly confirmed that the sequences of the two non-MOCV1/2 Swedish isolates correspond to MOCV3, and we matched the sequences generated to the restriction maps of various MOCV subtype variants. Our study showed that subtype variants and phylogenetic subgroups are equivalent terms, that these subtype variants/phylogenetic subgroups may have diverged from the prototype MOCV genotype sequences following large-scale recombination events, and that some of the recombinant segments may hint at the existence and partial sequence content of MOCV genotypes and subtype variants lacking complete genome sequences, specifically MOCV4 and the subtype variant MOCV1vb. Finally, we proposed that the determination of putative novel MOCV genotypes should be based on pairwise complete genome similarity while following the same genotype indexing as used in the pioneering molecular-epidemiological studies in the 1980s and 1990s.

## MATERIALS AND METHODS

### Complete MOCV genome sequences

All 19 previously generated and publicly available complete MOCV genome sequences were downloaded from the GenBank database: U60315 (11, 12), KY040274–KY040277 (13), MN202187 and MH646551 (45), and MH320547–MH320556 (14). In addition, in this study, a total of 47 novel complete MOCV genome sequences were assembled and functionally annotated, and they have been submitted to GenBank and GenBank TPA under the following accession numbers: MN931740–MN931752, BK0102005–BK0102038, OQ401159, and OQ401160.

### Sample origin and processing

The sequences MN931740–MN931752 were assembled from whole genome shotgun (WGS) sequencing reads conducted in the scope of this study. Whole DNA was isolated from molluscum contagiosum fresh-frozen tissue samples obtained by curettage using the QIAmp DNA Mini Kit (Qiagen, Hilden, Germany), as described previously (14). Sequencing library preparation and sequencing were contracted to GATC (GATC Biotech AG, Konstanz, Germany); libraries were prepared using the GATC in-house library preparation procedure, and sequencing reads were recorded using an Illumina HiSeq4000 (Illumina, San Diego, CA, USA) sequencing instrument in paired-end format (2 × 150 bp).

The sequences BK0102005–BK0102038 were assembled from WGS data (Illumina, California, USA; 2 × 150 bp) collected and deposited to the NCBI sequence read archives in the scope of an earlier study focusing on the skin metagenome in DOCK8-deficient immune-compromised patients, bioproject accession no. PRJNA471898 (46) (see Table S1 for SRR accession numbers and other details).

The sequence OQ401159 was assembled from single-end IonTorrent (IonTorrent PGM, ThermoFisher Scientific, Waltham, MA, USA) sequencing reads. The sequence OQ401160 was reassembled from single-end reads obtained using an IonTorrent S5 machine and Oxford Nanopore Technologies R10.4 High Quality sequencing reads (Oxford Nanopore Technologies plc—ONT, Oxford, UK) were obtained using an ONT GridIon device. DNA was isolated from lesion curettage samples, and shotgun libraries were prepared using the Ion Xpress Plus Fragment Library Kit for AB Library Builder System. The sequencing library for ONT sequencing was prepared using the Ligation Sequencing Kit V14 (SQK-LSK114, ONT) and loaded on the Flow cell FLO-MIN114 for sequencing; basecalling was performed using the Guppy basecaller (v6.5.7 , ONT).

### Complete MOCV genome sequence assembly and mutation calling

The complete MOCV genome sequences MN931740–MN931752 and BK0102005–BK0102038 were assembled *de novo* using SPAdes v3.12 (47, 48), with optimal assembly parameters tailored to each sample independently. Prior to assembly, reads were checked for sequencing adapter content and trimmed using Bbduk (Bbtools) (49, 50); after trimming, reads shorter than 100 bp were removed. Scaffolding was performed using Contiguator (51) with the reference sequences U60315 (MOCV1) and KY040274 (MOCV2). The complete MOCV genome sequence draft OQ401159 was assembled into contigs using spades v3.15.2 with the command line parameters: “–iontorrent” and “–k21,33,55,77,81,87,99,111”; the parameter “–cov-cutoff” was optimized to maximize contiguity. Scaffolding was performed using RagOut (52), and gaps were closed using Abyss-sealer (53). The complete genome sequence OQ401160 was scaffolded from high-quality ONT sequencing reads using the Flye assembler v2.9.2 (54), and the MOCV2 sequence KY040274 was used as a guide.

Assembled scaffolds were refined using Pilon v1.24 (55), and read-assembly mapping was performed using Bwa mem v0.7.17 (56) and processed using Samtools v1.9 (57). Genome completion was estimated by manually inspecting scaffold length (~180–200 kbp) and by the presence of inverted terminal repeat regions; genome identity (MOCV) was confirmed based on similarity to the MOCV reference sequences U60315 and KY040274. The sequence OQ401160 was refined using Pilon, with the combined alignment of ONT and IonTorrent reads.

Mutations versus the reference genome U60315 were found using Ivar (58), and the effects were annotated using SnpEff v5 (59). Pairwise genome alignments were carried out using Minimap2 (60) and processed using Samtools v1.9 (57).

### Functional annotation

The MOCV genome sequences MN931740–MN931752 and BK0102005–BK0102038 were functionally annotated by annotation transfer. We searched the refined genome scaffolds for ORFs using the NCBI tool ORFfinder (https://www.ncbi.nlm.nih.gov/orffinder/; parameters: “–ml 75 –s 0 –n true –strand both”). The ORFs were compared to the collection of known MOCV genes using blastp at the threshold identity level of 40%.

Functional annotation of sequence features in OQ401159 and OQ401160 was carried out in greater detail. Two separate feature tracks were obtained and curated manually. The final track consisted of the union of the two curated feature tracks. One feature track was obtained with Prokka (61), using the parameters “–kingdom Viruses –rnammer” and keeping “ATG” as the only plausible start-codon. The second track consisted of features resembling poxviral non-MOCV genes, it was obtained by searching ORFs (as described above) versus the GenBank database of protein amino acid sequences for the taxonomic family *Poxviridae* using blastp. The tracks were searched for sequence domains translating into known proteins of MOCV or other poxviruses, which we tried to encode into single CDS and gene features. In some cases, two or more CDS regions showed sequence similarity to distinct parts of a known poxviral protein, and we were unable to discern whether this was due to technically introduced artifacts. In these cases, gene features were constructed on top of the several CDS features resembling a single database protein.

### MOCV genome endonuclease restriction fragment size profiles: MOCV types and subtypes

Reference *BamHI*, *ClaI*, and *HindIII* (restriction enzymes) restriction fragment size profiles (restriction profiles) of MOCV types (MOCV1–4) and MOCV1 subtypes (MOCV1p, MOCV1va, MOCV1vb1, MOCV1vb2, and MOCV1vc) were derived from and encoded based on relevant literature (18, 19, 44).

The MOCV genomes were sought for restriction enzyme sequence recognition patterns (*BamHI:* GGATCC, *ClaI*: ATCGAT, and *HindIII*: AAGCTT) and fragmented *in silico* according to the positions identified. These simulated restriction profiles for each of the restriction enzymes were plotted side by side with the reference restriction profiles to ease comparison. The analysis and visualization were carried out using custom Python (v3.6) scripts depending on the Python modules NumPy (v1.19.5), SciPy (v1.5.3), Matplotlib (v3.3.3) (62), and BioPython (v1.78).

### Phylogenomic analyses, detection of recombination, and genotyping

Multiple nucleotide sequence alignments were generated using Mafft (v7.310) (63). Phylogenetic analyses were carried out using IQ-TREE v2 (v2.2) (30, 31); substitution models were selected using ModelFinder (64), and 1,000 permutations were considered in Ufbootstrap support score calculations (32). The phylogenetic trees were visualized using FigTree (v1.4; http://tree.bio.ed.ac.uk/software/figtree/).

Potentially recombinant sequence segments were elucidated as described previously (14). In brief, recombinant segment candidates were sought using silhouette coefficient (65, 66) analysis performed on the complete genome sequence alignment and along it in sequence kernels using a kernel length of 3,000 bp and a kernel step of 300 bp, with the reference classes based on the phylogenetic grouping of complete MOCV genomes with a sequence similarity threshold >98%. Boundary coordinates of the recombinant sequence segments (recombination breakpoints) were determined using the Recombination Detection Program (RDP5) (34); for confirmation, individual phylogenetic trees were drawn for each decoupled putative recombinant sequence segment and compared to the tree of the non-recombinant part of the complete genome sequences.

## Data Availability

All novel complete MOCV genome sequences characterized in this study were deposited with NCBI. For accession numbers, see Table S1.

## SUPPLEMENTAL MATERIAL

Supplemental figures mbio.02224-23-s0001.pdf Fig. S1 to S3.

Table S1 mbio.02224-23-s0002.xlsx Overview information of the molluscum contagiosum virus genomes included, including GenBank sequence accession numbers and elementary genome sequence statistics.

Table S2 mbio.02224-23-s0003.xlsx Pairwise sequence similarities between the molluscum contagiosum virus genomes across various stratifications.

Table S3 mbio.02224-23-s0004.xlsx Genomic coordinates of restriction enzyme recognition sites and recombinant sequence segments.

Table S4 mbio.02224-23-s0005.xlsx Novel putative functional features in the MOCV3 sequences OQ401159 and OQ401160: common ORFs, fragmented ORFs, and ORFs predicted *de novo*.

Table S5 mbio.02224-23-s0006.xlsx Mutations in the updated collection of the complete MOCV genome sequences.

ASM does not own the copyrights to Supplemental Material that may be linked to, or accessed through, an article. The authors have granted ASM a non-exclusive, world-wide license to publish the Supplemental Material files. Please contact the corresponding author directly for reuse.
