# Supplementary material for: Comprehensive analysis of 66 complete molluscum contagiosum virus (MOCV) genomes: characterization and functional annotation of 47 novel complete MOCV genomes, including the first genome of MOCV genotype 3, and a proposal for harmonized MOCV genotyping indexing
Source: mBio. 2023 Nov 10;14(6):e02224-23. doi: 10.1128/mbio.02224-23 (PMC10746250; doi:10.1128/mbio.02224-23)
Supplement: Supplemental figures — Fig. S1 to S3. [file mbio.02224-23-s0001.pdf]

# Comprehensive analysis of 66 complete molluscum contagiosum virus (MOCV) genomes: characterization and functional annotation of 47 novel complete MOCV genomes, including the first genome of MOCV genotype 3, and a proposal for harmonized MOCV genotyping indexing

Tomaž Mark Zorec,[a] Erik Alm,[b] Maria Lind Karlberg,[b] Reza Advani,[b] Lea Hošnjak,[a] and Mario Poljak[1]#

[a]Laboratory for Molecular Microbiology and Slovenian HIV/AIDS Reference Center, Institute of Microbiology and Immunology, Faculty of Medicine, University of Ljubljana, Zaloška 4, Ljubljana, Slovenia

[b]Department of Microbiology, Public Health Agency of Sweden, Solna, Sweden

#Address correspondence to Mario Poljak (mario.poljak@mf.uni-lj.si)

## Appendices

# Appendix A: Supplementary Tables

**Supplementary Table S1. Overview information of the molluscum contagiosum virus genomes included, including GenBank sequence accession numbers and elementary genome sequence statistics.** Key: GC = proportion of guanosine and cytosine, Hs = Hamming similarity, No. Gene = number of gene features, No. CDS = number of CDS features, N = number of valid pairwise distances.

**Supplementary Table S2. Pairwise sequence similarities between the molluscum contagiosum virus genomes across various stratifications.**

**Supplementary Table S3. Genomic coordinates of restriction enzyme recognition sites and recombinant sequence segments.**

**Supplementary Table S4. Novel putative functional features in the MOCV3 sequences OQ401159 and OQ401160: common ORFs, fragmented ORFs, ORFs predicted *de novo.***

**Supplementary Table S5. Mutations in the updated collection of the complete MOCV genome sequences.** Mutations were called and annotated based on pairwise sequence alignments with the MOCV1 sequence U60315 using Minimap2, Samtools, and SnpEff.

# Appendix B: Supplementary Figures

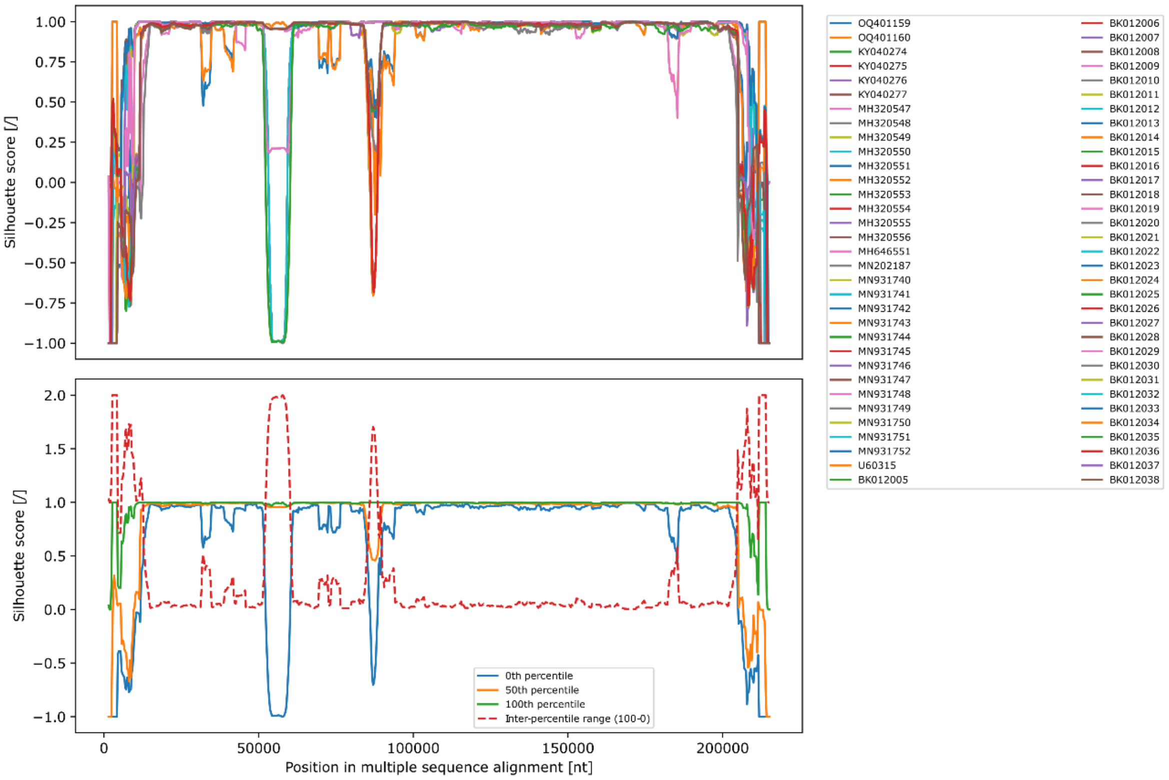


**Supplementary Figure S1. Sliding kernel-based analysis of silhouette coefficients along the complete molluscum contagiosum virus (MOCV) genome sequence alignment.** To quantify local disagreement with the global phylogenomic MOCV, sequence grouping silhouette coefficients (1) were calculated along each of the MOCV genomes in sequence kernels of 3,000 bp with a step size of 300 bp **(above).** Percentiles were calculated over values of all included MOCV genomes at every position-kernel. Traces of the 0th, 50th, and 100th percentile, and the interpercentile range between the 0th and the 100th percentile were plotted along the length of the genomes to aid in identification of recombinant sequence segments **(below).** Candidate sequence regions were subject to further analysis of recombination, specifically breakpoint placement and phylogenetic contrasting. The analysis was carried out using Python 3.8, silhouette coefficient calculations were facilitated by the Python module scikit-learn (1), and the plots were visualized using matplotlib (2).

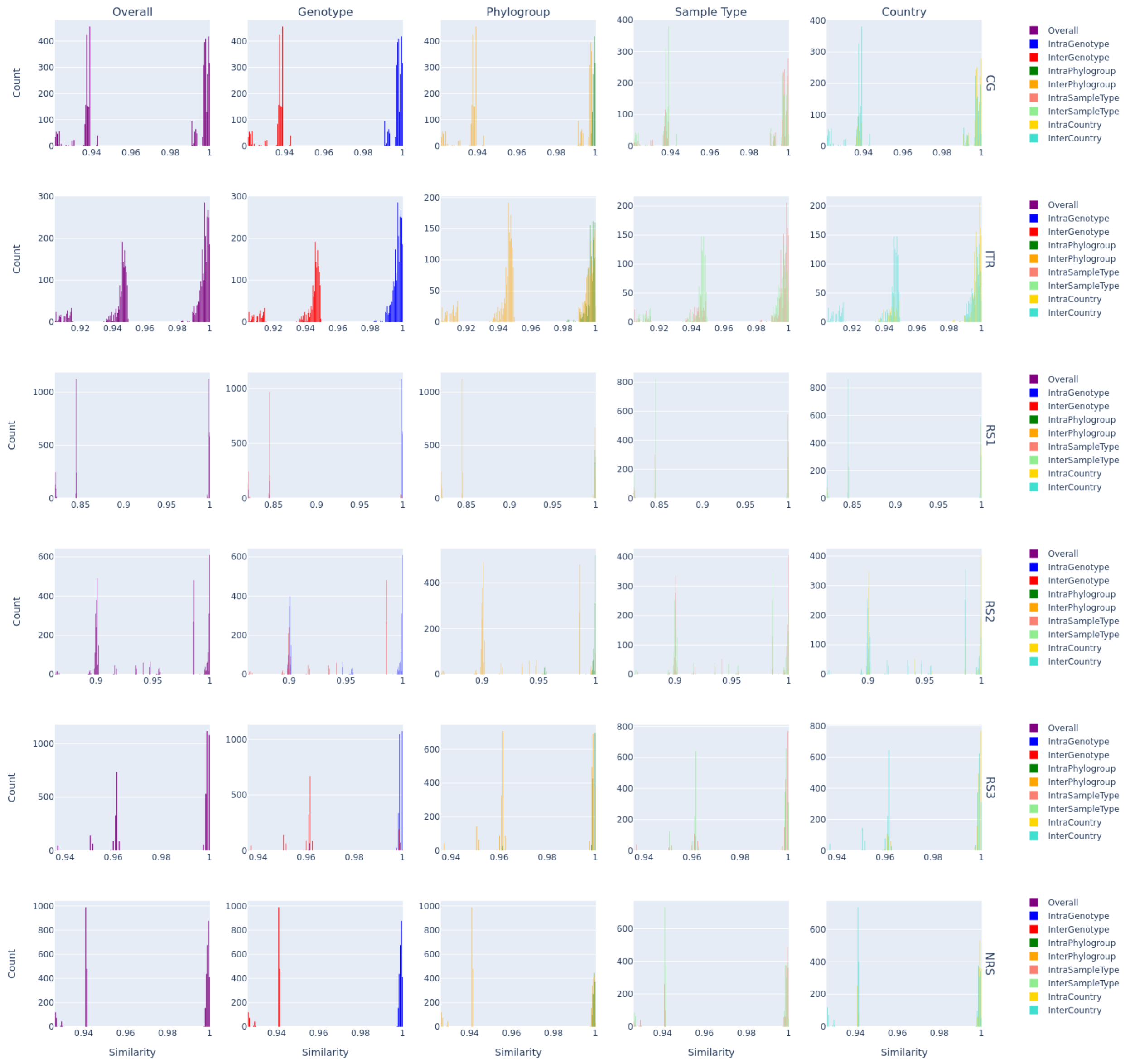


**Supplementary Figure S2. Pairwise sequence similarity histograms.** Different data stratifications (intra/inter-genotype, intra/inter-phylogroup, intra/inter–sample type, and intra/inter–country of origin) are color-coded and given in separate columns. Different sequence contexts, the complete genome sequences, recombinant segments, the inverted terminal repeat regions, and concatenated non-recombinant genome sequence regions are given in rows. The plots were produced using the Python 3.8 module Plotly Express.

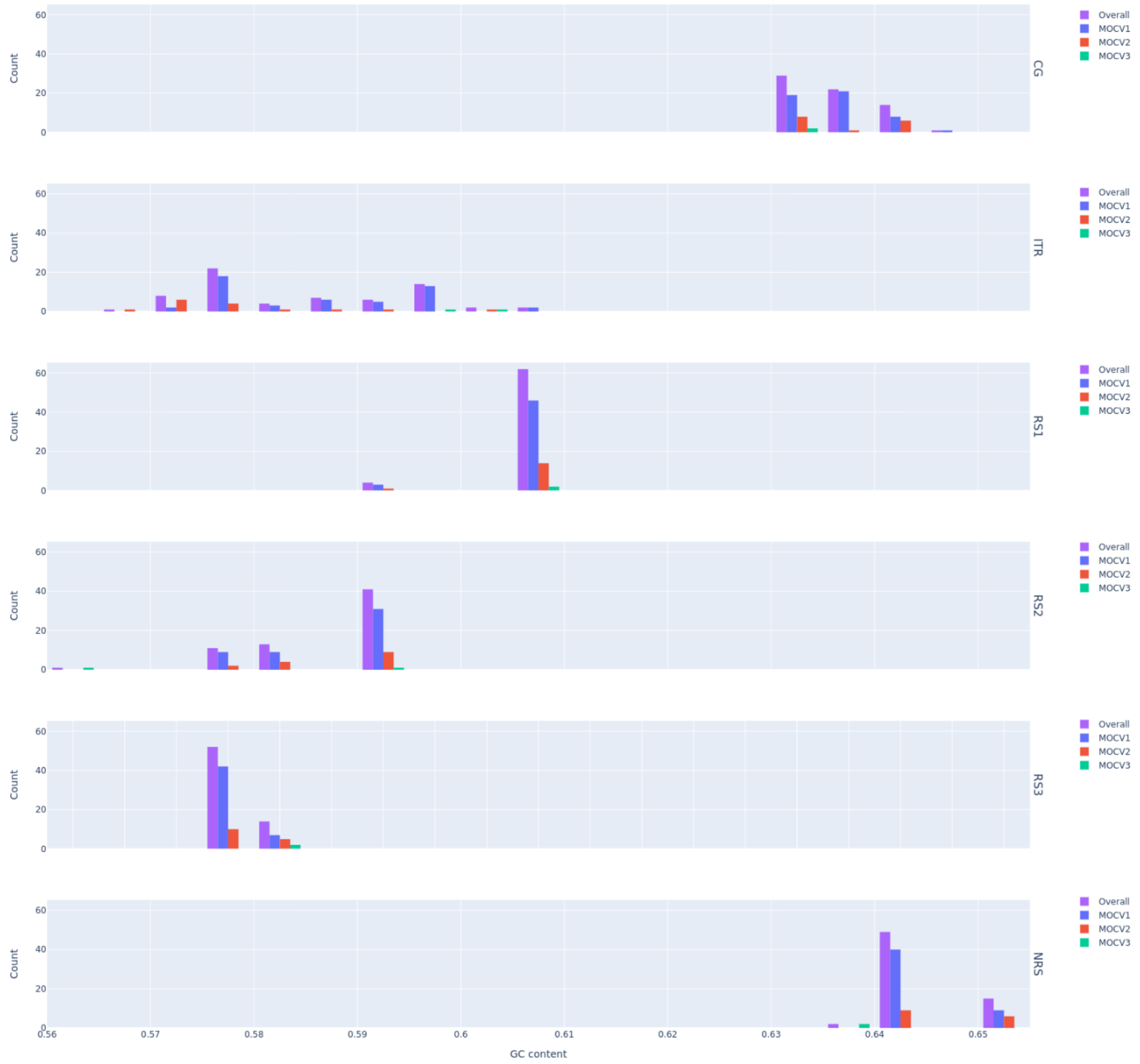


**Supplementary Figure S3. GC content histograms complete MOCV genome sequences, the recombinant and non-recombinant sequence segments, and the inverted terminal repeats (rows).** Histograms illustrate the overall distributions of GC content and for each of the three MOCV genotypes (color-coded). The plots were produced using the Python 3.8 module Plotly Express.

# References


1. Pedregosa F, Varoquaux G, Gramfort A, Michel V, Thirion B, Grisel O, Blondel M, Prettenhofer P, Weiss R, Dubourg V, Vanderplas J, Passos A, Cournapeau D, Brucher M, Perrot M, Duchesnay E. 2011. Scikit-learn: Machine Learning in Python. J Mach Learn Res 12:2825–2830.
2. 2. Hunter JD. 2007. Matplotlib: A 2D graphics environment. Comput Sci Eng 9:90–95.
